# Prenatal Opioid Exposure and Intermittent Hypoxemia in Preterm Infants: A Retrospective Assessment

**DOI:** 10.3389/fped.2017.00253

**Published:** 2017-12-06

**Authors:** Elie G. Abu Jawdeh, Philip M. Westgate, Amrita Pant, Audra L. Stacy, Divya Mamilla, Aayush Gabrani, Abhijit Patwardhan, Henrietta S. Bada, Peter Giannone

**Affiliations:** ^1^Division of Neonatology, Department of Pediatrics, University of Kentucky, Lexington, KY, United States; ^2^Department of Biostatistics, College of Public Health, University of Kentucky, Lexington, KY, United States; ^3^College of Medicine, University of Kentucky, Lexington, KY, United States; ^4^Children’s Hospital of Michigan, Detroit, MI, United States; ^5^Department of Pediatrics, New Jersey Medical School, Newark, NJ, United States; ^6^Department of Biomedical Engineering, College of Engineering, University of Kentucky, Lexington, KY, United States

**Keywords:** prenatal, opioid exposure, opiates, intermittent hypoxemia, apnea, preterm infants

## Abstract

**Introduction:**

Intermittent hypoxemia (IH) is defined as episodic drops in oxygen saturation (SpO_2_). Preterm infants are at increased risk for IH due to their immature respiratory control/apnea of prematurity. The clinical relevance of IH is a relatively new observation with rising evidence linking IH to neonatal morbidities and long-term impairment. Hence, assessing factors that influence IH in preterm infants is imperative. Given the epidemic of opioid misuse in the USA, there is an urgent need to understand the impact of prenatal opioid exposure on neonatal outcomes. Hence, we wanted to assess the relationship between isolated prenatal opioid exposure and IH in preterm infants.

**Methods:**

In order to accurately calculate IH, SpO_2_ data were prospectively collected using high-resolution pulse oximeters during the first 8 weeks of life in preterm infants less than 30 weeks gestational age. Data related to prenatal opioid misuse were retrospectively collected from medical charts. Infants with tobacco or poly-drug exposure were excluded. The primary outcome measure is percent time spent with SpO_2_ below 80% (%time-SpO_2_ < 80). The secondary outcome measure is the number of severe IH events/week with SpO_2_ less than 80% (IH-SpO_2_ < 80).

**Results:**

A total of 82 infants with isolated opioid exposure (*n* = 14) or who were unexposed (*n* = 68) were included. There were no significant differences in baseline characteristics between opioid exposed and unexposed groups. There was a statistically significant increase of 0.23 (95% CI: 0.03, 0.43, *p* = 0.03) in mean of the square root of %time-SpO_2_ < 80. The number of IH-SpO_2_ < 80 events was higher in the opioid exposed group (mean difference = 2.95, 95% CI: −0.35, 6.25, *p*-value = 0.08), although statistical significance was not quite attained.

**Conclusion:**

This study shows that preterm infants prenatally exposed to opioids have increased IH measures compared to unexposed infants. Interestingly, the increased IH in the opioid exposed group persists beyond the immediate postnatal period.

## Introduction

Intermittent hypoxemia (IH) is defined as brief, episodic drops in oxygen saturation (SpO_2_) (1, 2). Preterm infants are at increased risk for IH due to their respiratory control instability/apnea of prematurity superimposed on immature lung structure/function. IH in preterm infants can persist beyond discharge from the neonatal intensive care unit (NICU) (3). Brief episodes of oxygen desaturations may seem clinically insignificant, but these IH episodes, occurring up to hundreds of times per day, have a cumulative effect on neonatal morbidity and mortality. There is ample evidence showing a significant effect of IH on neurocognitive handicap, decreased neuronal integrity, increased inflammation and oxidative stress, and impaired growth (4, 5). Furthermore, IH has been linked to severe retinopathy of prematurity and long-term neurodevelopmental impairment such as worse language and motor outcomes (2, 6–9). The clinical relevance of IH is a relatively new observation with the advent of high-resolution pulse oximeters and assessing factors that influence IH is imperative.

There is a rise in substance misuse in the USA reaching a nationwide epidemic (10–15). There is an urgent need to understand the impact of prenatal opioid exposure on neonatal outcomes (5). Opioid exposure is associated with long-term neurobehavioral and developmental impairment in infants (16–23). Opioids are known to suppress breathing and respiratory effort especially in neonates (24). Since most mothers who misuse opioids have also been found to smoke and use poly-drugs that affect breathing pattern, it has been challenging to assess the isolated effect of prenatal opioid exposure on respiratory outcomes. Prenatal tobacco exposure alters respiratory control and worsens lung function (25–29). Prenatal exposure to other illicit drugs such as cocaine perturbs maturation of respiratory control, resulting in disruption of postnatal respiration (30). Only few studies were able to assess the effect of isolated opioid exposure on neonatal respiratory outcomes. However, these studies included mostly later preterm and term infants or were limited to short monitoring times and small sample sizes (31, 32). In this study, we utilize continuous high-resolution pulse oximeters to assess the relationship between isolated prenatal opioid exposure and IH in preterm infants during the first 2 months of life.

## Materials and Methods

### Study Design and Data Collection

Oxygen saturation data were prospectively collected from 130 preterm infants less than 30 weeks gestational age (GA) admitted to our level 4 NICU between November 2014 and April 2017. We used high-resolution pulse oximeters (Radical 7: Masimo, Irvine, CA, USA) set at 2 s averaging time and 1 Hz sampling rate to continuously monitor patients during the first 8 weeks of life. In order to differentiate intermittent from sustained hypoxemia, we included events between 4 and 180 s (1). The exact threshold below which IH is clinically significant is controversial. A drop in SpO_2_ to less than 80% is widely considered to be clinically relevant (1, 2, 6). Therefore, the primary outcome measure was defined as percent time spent with SpO_2_ below 80% (%time-SpO_2_ < 80). The secondary outcome measure was defined as the number of severe IH events with SpO_2_ less than 80% (IH-SpO_2_ < 80). Other outcome measures such as length of stay and neonatal morbidities were collected.

Pulse oximeters were equipped with serial data recorders (Acumen Instruments Corp.) for continuous data collection. Novel programs were utilized to filter and analyze data (Matlab, Natick, MA, USA) (1, 33). Data with artifacts were excluded. Only SpO_2_ data with good signal were included in the analyses. Preterm infants less than 30 weeks GA were included. Infants with major congenital malformations were excluded.

Data related to substance misuse and tobacco use were retrospectively collected from medical charts. If a mother chronically used prenatal opioids and/or the maternal/neonatal drug screens were positive for opioids, then the infant was considered for screening. Infants were then excluded from the study if the mother used tobacco, alcohol, or other drugs (such as cannabis); i.e., in order to assess for isolated opioid exposure, patients with any other exposure were excluded. Infants in our cohort who were not exposed to opioids, tobacco, or other drugs served as controls. Neonatal meconium or urine drug screens are performed in the immediate newborn period. Positive drug screens due to opioids and other medications used for pain or sedation during delivery were excluded, as they do not represent prenatal misuse. Tobacco and alcohol use were collected from mothers’ medical records, as the toxicology screens at our hospital do not test for alcohol or tobacco exposure. The study was approved by the University of Kentucky Institutional Review Board, and informed consent was obtained prior to SpO_2_ data acquisition.

### Statistical Analysis

Descriptive statistics for continuous variables are presented as either the mean with SD or median with interquartile range, and frequencies and percentages are given for categorical variables. Two-sample *t*-tests and Wilcoxon two-sample tests were used to compare opioid exposure to non-exposure with respect to continuous variables, and chi-square or Fisher’s exact tests were used for categorical variables. To compare opioid exposure to non-exposure with respect to IH measures over time, we utilized multivariate Gaussian linear modeling in order to account for repeated measurements from subjects, and to adjust for the potential confounders of GA, birth weight, APGAR score at 5 min of life, gender, and the use of prenatal steroids. In order to meet statistical assumptions in these models, the square root of the IH measures was taken. Furthermore, weekly observations were weighted by the percentage of time IH was tracked during the given week. Analyses were conducted in SAS version 9.4 (SAS Institute, Cary, NC, USA), and all tests were two-sided with a 5% significance level.

## Results

**Figure 1:**
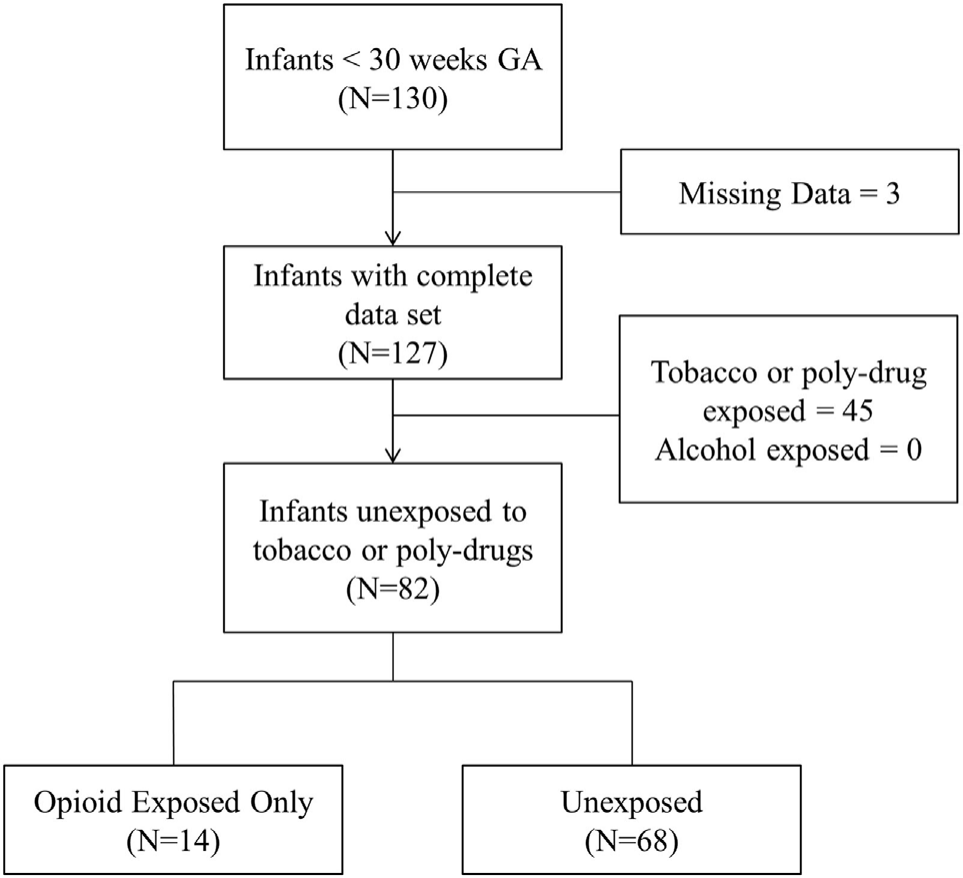
Flow diagram for patient eligibility.

Of the 127 infants in our database with complete data sets, 19.7, 29.1, and 4.7% were prenatally exposed to opioids, tobacco, and cannabis, respectively. None were exposed to alcohol, cocaine, and other illicit drugs. Opioid exposed infants were positive for buprenorphine metabolites (64%), oxycodone (16%), and other opioids such as heroin and fentanyl (20%). A total of 82 infants qualified for analysis as they were either unexposed to any illicit drug/tobacco (*n* = 68) or exposed to opioids only (*n* = 14). Figure 1 presents the flow diagram for patient eligibility and exclusion.

There were no significant differences in baseline characteristics as presented in Table 1. The mean GA was 27 weeks in both groups. There were no significant differences in birth weight, gender, and Apgar scores at 5 min of life. The vast majority of infants received prenatal steroids with no difference between groups. There were no significant differences in respiratory outcomes and neonatal morbidities between groups as presented in Table 2. Our cohort included preterm infants less than 30 weeks GA. Essentially all infants had respiratory distress syndrome and received surfactant. Severe bronchopulmonary dysplasia, postnatal steroids use for lung disease, and oxygen need at 28 days, 36 weeks postmenstrual age, and at discharge did not differ between opioid exposed and unexposed groups (all *p* = NS). Other neonatal morbidities such as patent ductus arteriosus, late onset sepsis, and necrotizing enterocolitis did not differ between groups (all *p* = NS). None of the exposed infants died versus nine deaths in the unexposed group (*p* = 0.35). The median length of stay was 17 days longer in the opioid group (85 days) compared to unexposed group (68 days); however, the results were not statistically significant (*p* = 0.32).

**Table 1:** Baseline characteristics.

	Opioid exposed	Unexposed	*p*-Value
	
	*N* = 14	*N* = 68
Gestational age (weeks)	27.0 ± 2.1	27.0 ± 1.6	0.97
Birth weight (g)	948 ± 263	928 ± 247	0.79
Male	6 (43%)	23 (34%)	0.54
Apgar 5 min	7 (6, 7.5)	6 (5, 7)	0.21
Prenatal steroids	12 (86%)	61 (91%)	0.62

*Mean ± SD, median (interquartile range)*.

**Table 2:** Neonatal morbidities and outcomes.

	Opioid exposed	Unexposed	*p*-Value
	
	*N* = 14	*N* = 68
Received surfactant	14 (100%)	62 (91%)	0.58
Respiratory distress syndrome	14 (100%)	67 (99%)	1
Oxygen at 28 days of life	10 (71%)	39 (57%)	1
Oxygen at 36 weeks corrected age	7 (50%)	19 (28%)	0.26
Oxygen at discharge	9 (64%)	30 (44%)	0.18
Severe bronchopulmonary dysplasia	9 (64%)	27 (46%)	0.21
Postnatal steroids use for lung disease	6 (43%)	19 (29%)	0.35
Pneumothorax	1 (7%)	2 (3%)	0.43
Patent ductus arteriosus	8 (57%)	24 (35%)	0.13
Necrotizing enterocolitis	0 (0%)	2 (3%)	1
Late onset sepsis	3 (21%)	9 (13%)	0.43
Mortality	0 (0%)	9 (13%)	0.35
Length of stay (days)	85 (59, 101)	68 (56, 91)	0.32

*Frequency (%), median (interquartile range)*.

**Figure 2:**
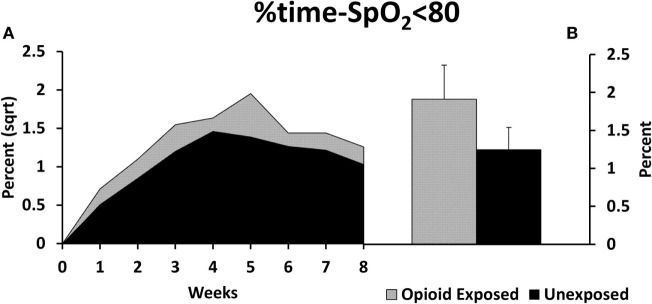
**(A)** Preterm infants exposed to prenatal opioids had increased time spent with oxygen saturation less than 80% (%time-SpO_2_ < 80) compared to unexposed infants (*p* = 0.03). The model adjusted for gestational age, birth weight, gender, prenatal steroids, and Apgar scores at 5 min of life. **(B)** This figure demonstrates the estimated average %time-SpO_2_ < 80 medians in both groups calculated using the adjusted model results. Sqrt, square root.

**Figure 3:**
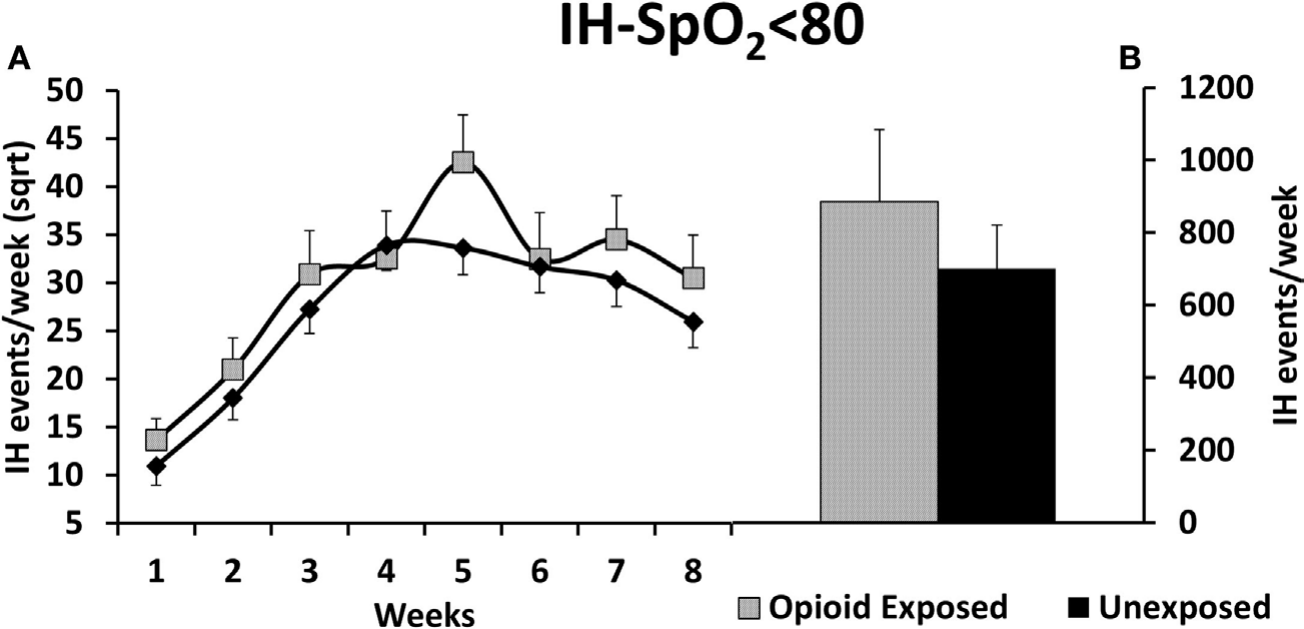
**(A)** Preterm infants exposed to prenatal opioids did not have a significant increase in number of intermittent hypoxemia (IH) events per week (IH-SpO_2_ < 80) compared to unexposed infants (*p* = 0.08). The model adjusted for gestational age, birth weight, gender, prenatal steroids, and Apgar scores at 5 min of life. **(B)** This figure demonstrates the estimated average IH-SpO_2_ < 80 medians of opioid exposed versus unexposed preterm infants calculated using the adjusted model results. Sqrt, square root.

There was a statistically significant increase in our primary outcome measure, %time-SpO_2_ < 80, as represented in Figure 2. The estimated difference in the means of the square root of %time-SpO_2_ < 80 was 0.23 (95% CI: 0.03, 0.43, *p* = 0.03). The mean number of IH events was estimated to be 2.95 (95% CI: −0.35, 6.25, *p*-value = 0.08) higher in the opioid exposed group, as represented in Figure 3; however, this did not reach statistical significance. Note that these results represent the square root of means in order to meet statistical assumptions in these models; estimated medians for IH measures are calculated using our model results and are presented in Figures 2B and 3B. Given increased death in the unexposed group, we then analyzed data excluding deaths, and results were similar. Specifically, there was a statistically significant increase in our primary outcome measure (%time-SpO_2_ < 80) in the opioid exposed compared to the unexposed group, with an estimated mean difference (square root) of 0.24 (95% CI: 0.05, 0.44, *p*-value = 0.02). Furthermore, the mean number of IH events was estimated to be 2.98 (95% CI: −0.20, 6.16, *p*-value = 0.07) higher in the opioid exposed group, not quite reaching statistical significance.

## Discussion

These results suggest that prenatal opioid exposure is associated with increased IH measures compared to unexposed preterm infants. This study has two main findings. First, interestingly, the increased IH measures in opioid exposed infants persisted beyond the early postnatal period. Preterm infants were continuously monitored with high-resolution pulse oximeters during the first 2 months of life. Second, we had the unique opportunity to assess the relationship between isolated opioid exposure and respiratory instability in preterm infants. It was challenging in the past to assess the relationship between isolated prenatal opioid exposure and respiratory outcomes/IH, as the majority of women who use opioids also smoke or misuse poly-drugs. Given our cohort demographics, we had the ability to report this association in infants exposed to opioids only.

Another interesting secondary finding in our study is the steady increase in IH in the first month of life before plateauing and then decreasing. This natural progression of IH has been described before from another cohort of preterm infants less than 28 weeks GA (1, 2). Our study replicates this finding from a new cohort of preterm infants less than 30 weeks GA. The rise in IH may be related to peripheral chemoreceptor dysregulation and development of lung disease (34).

Patients in our opioid exposed and unexposed groups did not significantly vary in terms of baseline characteristics (such as age, weight, gender) and neonatal morbidities (such as lung disease, patent ductus arteriosus, late onset sepsis, and necrotizing enterocolitis). In addition, we adjusted in the model for factors that may influence oxygenation in preterm infants such as GA and prenatal steroids. The finding of nine deaths in the unexposed group compared to no deaths in the opioid exposed group may be due to chance. Secondary analyses excluding deaths showed similar results with increased IH in the opioid exposed group. A significant secondary finding in this study is the high prevalence of tobacco and drug exposure in our cohort of preterm infants. The frequency of opioid exposure in our preterm population is higher than previously reported, thus creating urgency toward addressing this significant problem in this vulnerable patient population (10, 12–15).

There are multiple proposed mechanisms by which prenatal opioid exposure may affect breathing patterns and subsequent persistent IH in preterm infants. Prenatal opioid exposure alters the response to carbon dioxide and depresses central respiratory control centers (31, 35–38); a main driver for respiratory output. Olsen and Lees demonstrated a blunted response to carbon dioxide in methadone exposed infants compared to controls (31). Ali et al. compared the response to hypercarbia among three groups of term patients who were exposed to tobacco/substance misuse, tobacco alone, and unexposed controls. The authors showed a lower increase in central respiratory drive in response to hypercarbia in infants exposed to substance misuse as compared to tobacco alone and unexposed controls (35). Another mechanism that explains our results may be related to *in utero* hypoxia related to opioids. Prenatal opioids, especially street heroin, cause chronic intrauterine hypoxia leading to brainstem gliosis, resulting in injury to the central respiratory network. This may lead to respiratory instability and subsequent IH (36). Finally, data from animal models showed that exposure to opioid agonists caused downregulation of placental neurotransmitter receptors (39). Abnormalities or depletion of receptor sites, especially if the same process occurs in the fetal brain, could impair the function of the normal neonatal respiratory control network leading to frequent or prolonged apnea and subsequent IH.

Many studies have assessed the impact of prenatal opioid exposure on sudden infant death syndrome (SIDS) in infants with controversial results. This study does not address SIDS; rather, it focuses on IH, the end result of apnea of prematurity. However, the mechanism by which prenatal opioid exposure is associated with increased SIDS and IH may be similar. Although our study period focused on the inpatient setting, it is plausible that opioid exposed infants continue to have increased cardiorespiratory events/IH after discharge. Interestingly, compared to unexposed infants, opioid exposed infants had a trend toward longer length of stay (68 versus 85 days, *p* = NS), which may be related, in part, to persistent cardiorespiratory events.

A major limitation of this study is that data related to exposure were retrospectively collected. Another limitation is a lack of reporting daily caffeine use and daily respiratory support settings. At our center, virtually all infants with GA less than 30 weeks are started on caffeine therapy. Furthermore, our study focused on IH events and lacked reporting of apnea and bradycardia events. Lack of addressing heart rate is a limitation since bradycardia events may be associated with poor long-term outcomes (6). Another limitation is the small sample size; however, our sample size of isolated opioid exposure is relatively large compared to existing literature. This is a single center study; hence, our results may not be generalizable. Finally, we did not compare the long-term neurodevelopmental outcomes for exposed versus unexposed infants.

## Conclusion

There is rising evidence linking IH to neonatal morbidities and impairment. However, the exact threshold (frequency, duration, severity) by which IH leads to injury in preterm infants needs further investigation; i.e., any increase in IH may be associated with impairment in preterm infants. Furthermore, there is a need to understand factors, such as prenatal opioid exposure, that may influence IH and subsequently increase neonatal morbidities. In this study, we show an association between prenatal opioid exposure and increased IH measures in preterm infants. Studies to address the relationship between opioid exposures, IH, and long-term neurodevelopmental outcomes are imperative. Given the rising epidemic of opioid misuse in the USA, understanding the relationship between opioid exposure, IH and long-term impairment is imperative. A larger prospective study aimed at understanding these relationships may have a direct impact on short- and long-term management of preterm infants.

## Ethics Statement

This study was carried out in accordance with the recommendations of University of Kentucky Institutional Review Board (IRB). A written informed consent was obtained from all subjects included in this study. All subjects gave written informed consent in accordance with the Declaration of Helsinki. The protocol was approved by the University of Kentucky IRB.

## Author Contributions

EA designed and conceptualized the study and was actively involved in the enrollment, data collection, as well as analysis and interpretation of the results. He drafted the initial manuscript and wrote the final manuscript as submitted. PW was involved in conceptualization of the study and performed the statistical analyses. He also critically reviewed the manuscript and made the final approval manuscript as submitted. AP, AS, DM, and AG were involved in the conceptualization of the study, data collection, as well as analysis and interpretation of the results. They critically reviewed the manuscript and approved the final manuscript as submitted. AP was involved in the conceptualization of the study, the data acquisition software development, as well as in the analysis and interpretation of the results. He critically reviewed the manuscript and approved the final manuscript as submitted. HB and PG were involved in the conceptualization of the study as well as analysis and interpretation of the results. They critically reviewed the manuscript and approved the final manuscript as submitted.

## Conflict of Interest Statement

The authors declare that the research was conducted in the absence of any commercial or financial relationships that could be construed as a potential conflict of interest.

## Abbreviations

GA, gestational age; IH, intermittent hypoxemia; NICU, neonatal intensive care unit; SIDS, sudden infant death syndrome; SpO_2_, oxygen saturation; THC, tetrahydrocannabinol.
